# Computer supported collaborative learning in a clerkship: an exploratory study on the relation of discussion activity and revision of critical appraisal papers

**DOI:** 10.1186/1472-6920-12-79

**Published:** 2012-08-20

**Authors:** Willem JM Koops, Cees PM van der Vleuten, Bas A de Leng, Luc HEH Snoeckx

**Affiliations:** 1 Department of Medical Education, MMC Academy, Máxima Medical Center, De Run 4600, 5500 MB, Veldhoven, the Netherlands; 2 Department of Educational Development and Research, Faculty of Health, Medicine & Life Sciences, Maastricht University, Universiteitssingel 60, P.O. Box 6200 MD, Maastricht, the Netherlands; 3 Department of physiology, Faculty of Health, Medicine and Life Sciences, Maastricht University, Universiteitssingel 50, P.O. Box 6200 MD, Maastricht, the Netherlands

## Abstract

**Background:**

Medical students in clerkship are continuously confronted with real and relevant patient problems. To support clinical problem solving skills, students perform a Critical Appraisal of a Topic (CAT) task, often resulting in a paper. Because such a paper may contain errors, students could profit from discussion with peers, leading to paper revision. Active peer discussion by a Computer Supported Collaborative Learning (CSCL) environment show positive medical students perceptions on subjective knowledge improvement. High students’ activity during discussions in a CSCL environment demonstrated higher task-focussed discussion reflecting higher levels of knowledge construction. However, it remains unclear whether high discussion activity influences students’ decisions revise their CAT paper. The aim of this research is to examine whether students who revise their critical appraisal papers after discussion in a CSCL environment show more task-focussed activity and discuss more intensively on critical appraisal topics than students who do not revise their papers.

**Methods:**

Forty-seven medical students, stratified in subgroups, participated in a structured asynchronous online discussion of individual written CAT papers on self-selected clinical problems. The discussion was structured by three critical appraisal topics. After the discussion, the students could revise their paper. For analysis purposes, all students’ postings were blinded and analysed by the investigator, unaware of students characteristics and whether or not the paper was revised. Postings were counted and analysed by an independent rater, Postings were assigned into outside activity, non-task-focussed activity or task-focussed activity. Additionally, postings were assigned to one of the three critical appraisal topics. Analysis results were compared by revised and unrevised papers.

**Results:**

Twenty-four papers (51.6%) were revised after the online discussion. The discussions of the revised papers showed significantly higher numbers of postings, more task-focussed activities, and more postings about the two critical appraisal topics: “appraisal of the selected article(s)”, and “relevant conclusion regarding the clinical problem”.

**Conclusion:**

A CSCL environment can support medical students in the execution and critical appraisal of authentic tasks in the clinical workplace. Revision of CAT papers appears to be related to discussions activity, more specifically reflecting high task-focussed activity of critical appraisal topics.

## Background

In the clinical phase of the medical curriculum, during a clerkship, students learn primarily in the authentic context of the workplace [1,2] and are continuously confronted with clinical problems. Students have a preference for learning from clinical problems in the workplace because these problems are real and relevant to them [3,4]. To train clinical problem solving skills, medical students often use critical appraisal [4-6], defined as: “The process of assessing and interpreting evidence (usually by published research) by systematically considering its validity (closeness to the truth), results and relevance to the individual’s work” [7,8]. A practical task here is a Critical Appraisal of a Topic (CAT). This CAT task requires a student to first formulate a clinical question relating to a clinical problem encountered in the workplace. Next, the literature is investigated for articles offering evidence with relevance to the problem. Then, the student has to appraise the evidence critically, in relation to the aetiology, diagnosis, prognosis, therapy and follow-up of the case in question, and to describe the evidence table. Finally, the student considers the value of the evidence and presents the conclusion related to the clinical problem concerned [8,9]. A CAT paper written by an individual student can be considered as a first draft which has not been subject to any review. Since a CAT paper may contain errors like those of fact, calculation and interpretation, students can profit from a thorough discussion of their CAT paper with peers. Irrespective of whether they decide to revise or not revise the CAT paper afterwards [9,10]. However, such a collaborative activity poses logistical problems, particularly when students are dispersed over different training locations. Part of a solution may be provided by a Computer Supported Collaborative Learning (CSCL) environment, enabling students to engage in a structured, asynchronous discussion, independent of place and time [11-17]. However, it has been shown that such collaborative activities do not automatically result in positive learning outcomes. The success of CSCL depends on, among other factors, the intensity of the online activity within groups and its results [18-20]. Research on the use of CSCL by university students has shown that a more intense activity during discussions is associated with high task-focussed discussion activity, reflecting specifically higher levels of knowledge construction [21]. In a recent study, we demonstrated that medical students perceived subjective (knowledge) improvement of their learning outcomes during asynchronous discussions of an authentic CAT task in a CSCL environment [22]. Although high activity during asynchronous discussions in a CSCL environment appears to be associated with high task-focussed activity, it remains unclear whether students’ discussion activity influences their decision to whether or not to revise the CAT paper. Furthermore, it is not clear whether high discussion activity on CAT topics influences students to revise their CAT paper.

In present study we hypothesized that students who revise their CAT paper after discussing its content with peers in a CSCL environment, conduct an extensive discussion, with more task-focussed activity, than students who do not revise their paper. Besides it is hypothesised that students who revise their CAT paper show more discussion activity on critical appraisal topics than students who do not revise their CAT paper. Thus, the first objective of present study was to examine whether students who revised their paper showed more task-focussed activity compared with students with unrevised papers. The second objective was to evaluate whether students who revised their paper showed more discussion activity on critical appraisal topics, compared with student with unrevised CAT papers.

This paper details the process of a peer discussion of a CAT paper on a clinical problem, and reports the effects on students activity during discussion in a CSCL environment.

## Methods

### Participants and task

Between January 2008 and June 2010, all sixth year students of the medical curriculum of the Faculty of Health, Medicine and Life Sciences, Maastricht University, the Netherlands participated in an eighteen-week clerkship in a discipline of their choice. The clerkships were offered in nine different hospitals, eight in the Netherlands and one in Austria. One of the tasks during the clerkship required students to investigate a self-selected clinical problem encountered during the elective and to write a pre-formatted critical appraisal of a topic (CAT) paper on it, a task with which students were familiar from ample earlier experience.

### Study design

**Figure 1:**
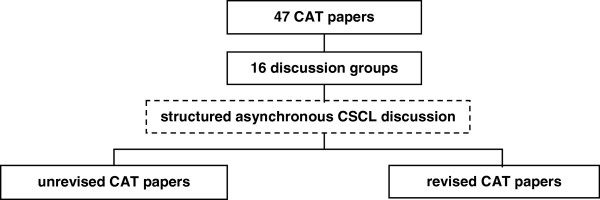
study design

Sixty-six medical students were invited by e-mail for this study, forty-seven of which voluntarily agreed to participate in the study. The participants received informed consent before the start of the study and were free to withdraw their cooperation at any time. They were randomly allocated into sixteen groups, fifteen groups of three and one group of two students. Each student uploaded his individual CAT paper to a ‘drop-box’, read the papers of the peers in his group and provided his comments in an asynchronous structured discussion forum in the open source CSCL environment DOKEOS ( http://www.dokeos.com). The discussion was moderated by the student who’s paper was subject of discussion. After the online discussion, students were given the opportunity to revise their paper (Figure 1). Students had only access to their own discussion forum. The discussion activity and postings were automatically filed in the CSCL environment.

In order to structure the discussion of the CAT papers, the students were asked to address three topics: (1) the selection of the clinical problem, the formulation of the clinical question and the process of the literature search, (2) the study design and the methods of the article(s) selected on the basis of the literature search and, (3) the evidence provided by the article(s) that could be used to address the clinical problem and relevant clinical conclusions regarding the clinical problem. To help students with the CSCL discussion task, they received, by e-mail, an instruction manual containing information about the design and use of the CSCL environment, and about the schedule for the discussion. Students were free to make arrangements in their discussion group regarding the sequence of CAT papers to be discussed, as long as each individual CAT paper was discussed within a two-week period. Students received a password and logon code to access the CSCL environment and could familiarise themselves with the environment before starting the actual task.

### Measurement instruments and statistical analysis

 1. Content analysis of students’ postings on collaborative problem solving activity

To identify the type of collaborative problem solving activity, content analysis of students’ postings was performed according to the validated Rainbow system [23]. This content analysis system has been developed for any educational discussion forum, but to our knowledge, has not been used in medical education before. According to the Rainbow system, there are seven categories of communicative interaction, which can be grouped into three collaborative problem solving activities, i.e., outside activity, non-task-focussed activity or task-focussed activity (Table 1). Regarding the group of task-focussed activity, the categories 5, 6 and 7 are considered to reflect the highest levels of knowledge construction, respectively. In the present study, all postings of students CAT paper discussions were blinded. Thus, the analysis of the postings was conducted by the investigator, unaware of student characteristics and whether or not the paper, which was subject to the discussion, was revised. 

**Table 1:** Rainbow system for content analysis; activity, category, and category definitions

**Content analysis system for collaborative problem solving activity [**[23]**]**		
**Activity**	**Category**	**Definition**
**Outside**	*1. Outside*	Any interaction that is not concerned with interacting in order to carry out the defined task, e.g., talk about last night’s party.
**Non-task-focussed**	*2. Social relation*	Interaction concerned with managing the students’ social relations with respect to the task, e.g., greeting, leave-taking, politeness.
	*3. Interaction management*	Interaction concerned with managing the interaction itself, e.g., coordination (who will speak and who will not), establishing contact, topic shifting.
**Task-focussed**	*4. Task management*	Management of the progression of the task itself, e.g., planning what is to be discussed, establishing whether problem is solved or not.
	*5. Opinions*	Interaction concerned with expressing opinions about the topic of discussion, e.g., beliefs, acceptances.
	*6. Argumentation*	Expression of (counter-) arguments directly related to a thesis, or theses themselves, e.g., requests for justification
	*7. Broaden and deepen*	Interaction concerned with (counter-)arguments linked to (counter-) arguments, argumentative relations and the meaning of arguments themselves, e.g., elaborations of arguments, definition.

In principle individual postings were considered as a unit of analysis. However, when a posting contained multiple activities it was split into different units of analysis, which were then coded separately [21,24]. All units of analysis were counted and descriptive statistics were calculated for the three collaborative problem solving activities, as well as for the seven categories.

 2. Content analysis of students’ postings on CAT topics of discussion.

To examine the discussion on the three critical appraisal topics as well as the corresponding CAT task elements (Table 2), all analysis units identified as either category 5., 6., or 7. of task-focussed activity (see higher) were labelled to one of the topics and their elements. Descriptive statistics were performed on the frequency of analysis unit per discussion topic, overall, as well as on revised and unrevised papers.

**Table 2:** Prescribed critical appraisal topics and elements of the CAT task

**Critical appraisal topics of discussion**	**CAT task elements**
(1) Literature search regarding clinical problem	Preparation for executing the literature search
	Strategy of the literature search
	Results of the literature search
(2) Appraisal of the selected article(s)	Study design
	Study method
	Study outcome
(3) Relevant conclusion regarding the clinical problem	Evidence table of appraised article(s)
	Relevant clinical conclusion regarding clinical problem

A Mann–Whitney U test for independent samples was performed to compare the analysis units of the revised and the unrevised papers, with regard to the three collaborative problem solving activities and their corresponding categories, and with regard to the three critical appraisal topics and their elements. *P* < .05 was considered statistically significant. CAT task elements were identified in either the revised or unrevised papers, and compared by a Chi-square test.

## Results

 1. Content analysis of students’ postings on collaborative problem solving activity.

A total of 1582 units of analysis was identified in students’ postings in the various discussion groups (Table 3). In the discussions of revised papers (n = 24), the number of analysis units was almost twice as high as in the unrevised paper discussions (n = 23) *(P < .001)*. Non-task-focussed activity was identified in discussions on revised papers (23.2%), as well as on unrevised papers (25.8%). Task-focussed activity was relatively high in both revised (73.9%), and unrevised paper discussions (69.5%), but, in absolute terms, the task-focussed activity in the revised paper discussions was double as high as in the unrevised paper discussions *(P < .000).* A statistically significant higher number of units in the category 5. (Opinions*; P < .000),* 6. (Argumentation*; P < .005*), and 7. (Broaden and Deepen; *P < .016)* was found in the revised paper discussions compared with the unrevised paper discussions. Effect sizes (Cohens ‘d) on these categories were large, with exception of category 7.

 2. Content analysis of students’ postings on CAT topics of discussion.

**Table 3:** Frequencies and descriptive statistics of discussion activity in the revised and unrevised paper discussions

	**analysis units of revised papers (n = 24)**		**analysis units of unrevised papers (n = 23)**		**Mann–Whitney U test**	**Effect size (Cohens d’)**
	**frequency**	**median (min.-max.)**	**frequency**	**median (min.-max.)**	***p*-value**	
**Collaborative problem solving activities** (total of category 1.-7.)	1028	36 (15–94)	554	20 (1–53)	***P*** **< .001**	**1*.08***
**Outside activity ***Category 1.*	29	1 (0–10)	26	1 (0–4)	*P* < .716	0.05
**Non-task-focussed activity** (total of category 2. & 3.)	239	7.5 (0–31)	143	6 (0–15)	*P* < .179	0.57
*Category 2. Social Relation*	189	5.5 (0–26)	96	3 (0–11)	*P* < .071	*0.68*
*Category 3. Interaction Management*	50	2 (0–19)	47	1 (0–7)	*P* < .610	0.04
**Task-focussed activity** (total of category 4.-7.)	760	29 (11–65)	385	15 (0–45)	***P*** **< .000**	***1.17***
*Category 4. Task Management*	64	2 (0–19)	28	1 (0–7)	*P* < .107	0.48
*Category 5. Opinions*	481	17.5 (10–40)	269	10 (0–30)	***P*** **< .000**	***1.04***
*Category 6. Argumentation*	186	7.5 (0–19)	80	4 (0–9)	***P*** **< .005**	***0.98***
*Category 7. Broaden and Deepen*	29	0 (0–5)	8	0 (0–5)	***P*** **< .016**	0.54

Analysis units of task-focussed activity relating to the three critical appraisal topics of discussion are presented in Table 4. For the topic 2 ‘appraisal of the selected article(s)’ and topic 3 ‘relevant conclusion regarding the clinical problem’, the number of units was significantly higher in the revised than in the unrevised paper discussions. Effect sizes (Cohens ‘d) on these topics were large, as well.

**Table 4:** Frequency of analysis units of revised and unrevised paper in critical appraisal topics of discussions

	**analysis units of revised papers (n = 24)**		**analysis units of unrevised papers (n = 23)**		**Mann–Whitney U test**	**Effect size (Cohens’d)**
**Critical appraisal topics of discussion**	**frequency**	**median (min-max)**	**frequency**	**median (min-max)**	***P*-value**	
(1) Literature search regarding the clinical problem	258	9.74 (2.75–30.16)	173	8 (0–22.43)	*P* < .070	0.56
(2) Appraisal of the selected article(s)	276	9.72 (1.41–32.64)	137	4.7 (0–15)	***P*** **< .007**	**0.86**
(3) Relevant conclusion regarding the clinical problem	162	6.5 (0–12.69)	47	1.6 (0–9.29)	***P*** **< .000**	**1.54**

The frequency and percentages of CAT task elements identified in either revised or unrevised paper discussion are presented in Table 5. Overall, CAT task elements were discussed more in the revised than in the unrevised papers. In the revised paper discussions, every prescribed CAT topic and corresponding CAT task element was identified, where in the unrevised CAT paper discussions, the elements of topic (2): ‘Study design’ and ‘Study outcome’ were not identified as elements under discussion. Significantly more critical appraisal topics of discussion identified in the revised paper were found in topic (1): ‘Preparation for executing literature search’, and ‘Strategy of literature search’, every CAT task element of topic (2), and in topic (3): ‘Relevant clinical conclusion regarding clinical problem’.

**Table 5:** CAT task elements of critical appraisal topics discussed in revised and unrevised papers

**Critical appraisal topics of discussion**	**CAT task elements identified in discussion**	**Revised papers N = 24**	**Unrevised papers N = 23**	**Chi-square *P*-value**
(1) Literature search regarding the clinical problem	Preparation for executing literature search	15	5	***P*** **< .005**
	Strategy of the literature search	24	12	***P*** **< .000**
	Results of the literature search	11	7	*P* < .278
(2) Appraisal of the selected article(s)	Study design	15	0	***P*** **< .000**
	Study population	24	11	***P*** **< .000**
	Study outcome	4	0	***P*** **< .041**
(3) Relevant conclusion regarding the clinical problem	Evidence table of appraised research	6	2	*P* < .137
	Relevant clinical conclusion regarding clinical problem	18	6	***P*** **< .001**

## Discussion

The results of the present study indicate that a Computer Supported Collaborative Learning environment can effectively support medical students to learn collaboratively during clinical clerkships. By the execution of an authentic task such as a critical appraisal of a relevant clinical problem, students are stimulated to critically discuss and revise their critical appraisal paper. Students’ paper revision seems to be associated with an increased activity during discussions with peers, and to be related to higher task-focussed discussion activity as well as a more intense discussion of critical appraisal topics.

The discussion of students who revise their CAT paper substantially differs from that of students who do not revise. Revised paper discussions are more extensive, social and task-focussed, reflecting both low and higher levels of knowledge construction. These results findings are consistent with results obtained with CSCL in university classroom environments and medical workplace, showing that both social interaction and task-orientation are typical for an active discussion leading to higher levels of knowledge construction [25,26].

Furthermore, students who revise their CAT paper after discussing its content with peers in a CSCL environment show more discussion activity on critical appraisal topics, with a strong focus on the CAT task elements: strategy of the literature search, and appraisal of the study population. Other elements identified under discussion in the majority of revised papers were: ‘preparation for executing literature search’; ‘study design’, and ‘relevant clinical conclusion regarding clinical problem’. A study among undergraduate medical students showed no differences in critical appraisal skills between students who received a computer-based learning session and students who attended classroom lectures [17]. However, two studies on medical students’ individual learning in on-line critical appraisal modules during clinical clerkships showed positive outcomes in favour of on-line learning. One study measured medical students’ pre-and post-test scores on an individual pre-determined critical appraisal task, and showed improvement in executing a search strategy and in appraise a study design [27], while another study compared the critical appraisal skills of medical students after on-line learning with those of students without intervention, and reported a higher quality of the literature search after on-line learning [28]. In the above-mentioned studies as well as in present study, students worked individually on a critical appraisal task. However, in present study students worked on a self-selected clinical problem extended with a collaborative discussion on their paper with peers. These differences in study design could have influenced the finding in present study that not only identical CAT task elements were identified, but moreover, even more critical appraisal elements were found.

A limitation of present study is the challenge to control all variables in an on-line collaborative discussion. By the design of a structured discussion task a certain control of variables is achieved. Despite of this structured discussion, it can not be excluded that students’ performance in course may have influenced them to revise their paper. However, this phenomenon likely has played a minor role since it concerned last year medical students with a comparable knowledge level. Moreover, all students participating in the present study performed several critical appraisals during the previous four years of the medical curriculum, and thus can be considered to be experienced in writing a CAT paper. Therefore, it was expected that participants may not have felt great urgency to discuss the task. It thus is remarkable that, even after intensive training the skill in performing a CAT, 51% of the students revised their paper after collaborative online discussion. Therefore, students can profit by a peer discussion of their papers, irrespective whether they revise or not revise their paper [9,10]. Besides the effect of discussion with peers, other factors could have influenced students to revise the CAT paper. First, since students participated voluntarily in this study they were probably highly motivated to discuss with peers. Secondly, it can be emphasized that motivation for discussion is high because this critical appraisal task was related to a self-selected, authentic clinical problem. Even though it cannot be excluded that the discussion itself could stimulate students to discuss.

Another limitation is that the content analysis was performed by one researcher. Since the analysis was conducted according to a structured and validated analysis system [23], it seemed safe to assume that the analysis was well executed.

Furthermore, it cannot be denied that the sample of participants was relatively small. However, high effect sizes reveal a high effect on the students’ activity in discussion on two levels of knowledge construction and on two critical appraisal topics of the discussion.

In CSCL research, much attention has been given to measuring outcomes in terms of cognition, skills, critical thinking and problem solving, but research on what influences student learning during discussions is scant. Further research on a larger scale could be useful to clarify the learning processes during discussions and to what extent these processes affect the interaction among students and knowledge construction in CSCL. A controlled study comparing students interaction between an intense and limited discussion could be an interesting intervention for further research. Furthermore, it could be interesting to further research the question whether implementing an on-line discussion could be applied as a framework to support students’ learning in existing courses.

## Conclusions

A Computer Supported Collaborative Learning environment can support medical students in critically appraising clinical problems encountered during learning in the workplace. An increase in activity during the discussions seems to be related to more task-focussed activities and more discussion of critical appraisal topics.

## Competing interests

The authors report no conflicts of interest. The authors alone are responsible for the content and writing of the paper.

## Authors’ contributions

WJMK started the study, wrote the research design, conducted the research, collected and analysed the data, and wrote the manuscript. CPMV discussed on the research design, and helped to draft the manuscript. BAL discussed on the research design, and helped to draft the manuscript. LHEHS discussed on the research design, and helped to analyse the data, and drafted the manuscript. All authors read and approved the final manuscript.

## Pre-publication history

The pre-publication history for this paper can be accessed here:

http://www.biomedcentral.com/1472-6920/12/79/prepub
